# Does Pseudohypoaldosteronism Mask the Diagnosis of Congenital Adrenal Hyperplasia?

**DOI:** 10.4274/jcrpe.369

**Published:** 2011-12-06

**Authors:** Sebahat Yılmaz Ağladıoğlu, Zehra Aycan, Havva Nur Peltek Kendirci, Nilgün Erkek, Veysel Nijat Baş

**Affiliations:** 1 Dr. Sami Ulus Obstetrics and Gynecology, Pediatric Health and Disease Training and Research Hospital, Clinics of Pediatric Endocrinology, Ankara, Turkey; 2 Dr. Sami Ulus Obstetrics and Gynecology, Pediatric Health and Disease Training and Research Hospital, Clinics of Pediatrics, Ankara, Turkey; +90 312 305 65 08sebahatyilmaz@yahoo.comDr. Sami Ulus Obstetrics and Gynecology, Pediatric Health and Disease Training and Research Hospital, Clinics of Pediatric Endocrinology, Ankara, Turkey

**Keywords:** Pseudohypoaldosteronism, congenital adrenal hyperplasia

## Abstract

Hyponatremia and hyperpotassemia occurring in the first few weeks of life primarily indicate aldosterone deficiency due to salt-losing congenital adrenal hyperplasia (SL-CAH), while mineralocorticoid deficiency and insensitivity are the main causes of hyponatremia and hyperpotassemia in older infants. Some patients who present with vomiting and poor sucking, who have hyponatremia and hyperpotassemia and are initially diagnosed as CAH, during follow-up, are found to suffer from pseudohypoaldosteronism (PHA). This situation has been reported several times before. The cases described here represent the opposite situation: they presented with hyponatremia and hyperpotassemia, thus PHA was considered as aldosterone levels were very high, but subsequent investigation and genetic analysis led to the diagnosis of SL-CAH.

**Conflict of interest:**None declared.

## INTRODUCTION

Hyperpotassemia together with severe hyponatremia is rare in infancy but important as it can be life-threatening. Congenital adrenal hyperplasia (CAH) should be considered first among adrenal diseases in the differential diagnosis of hyponatremia if no gastrointestinal salt loss is present. Adrenal hypoplasia, isolated aldosterone deficiency, drug effects and pseudohypoaldosteronism (PHA) are other conditions that should be kept in mind in the differential diagnosis (1).

A congenital renal anomaly can cause PHA due to a lack of response to aldosterone in the distal tubule in male infants under 3 months of age in the presence of obstructive uropathy, vesicoureteral reflux (VUR) and/or urinary tract infection (UTI) (2) and this can be confused with CAH. 

Compensated salt-losing CAH (SL-CAH) is accompanied by increased androgen production, inadequate cortisol production and also increased renin and aldosterone levels; serum electrolytes are normal in this condition (3). However, hyponatremia and hyperpotassemia may develop due to the lack of aldosterone effect in case of a  renal anomaly, VUR and/or UTI (4) and this condition is called transient secondary PHA. In such patients, PHA should be considered first if hyponatremia and hyperpotassemia are present despite very high levels of aldosterone. The serum aldosterone level is low in the type of CAH with hyponatremia and hyperpotassemia as there is no aldosterone synthesis.  

We present the cases of two patients who were seen at our hospital with severe hyponatremia and hyperpotassemia. 

A diagnosis of PHA was first considered due to the high aldosterone levels, but the ultimate diagnosis was CAH. These cases are reported to emphasize the importance of not missing CAH in patients presenting with a clinical picture of PHA.

## CASE REPORTS

**Patient 1**

A 45-day-old male baby born at term with a birth weight of 2600 g presented with vomiting and poor sucking. Body weight was 2600 g, indicating that the patient had not gained weight since birth. Physical examination revealed severe dehydration and mild scrotal hyperpigmentation. Laboratory results were as follows: Serum Na: 114 mEq/L (N: 135-143 mEq/L),  K: 7.7 mEq/L (N: 3.5-5.5mEq/L), blood pH: 7.3, HCO3: 12 mmol/L, BUN: 24 mg/dL (0-10 mg/dL), creatinine: 0.5 mg/dL (0.3-1.2 mg/dL). Urinalysis revealed leukocytes and urine culture grew 100 000 colonies/mL E. coli. Intravenous saline treatment was started together with antibiotics for the UTI. Hormonal evaluation results were adrenocorticotropic hormone (ACTH): 186 pg/mL (N: 3-46 pg/mL), basal cortisol: 8 μg/dL, renin: 836 pg/mL (N: 2.4-37 pg/mL) and aldosterone: 450 pg/mL (N: 20-700 pg/mL) - findings  which led to a preliminary diagnosis of PHA. A high ACTH value was noted. The ACTH stimulation test performed to rule out CAH gave the following results for 17-hydroxyprogesterone (17-OHP) response: 27.7 ng/mL at 0 time, 37.2 ng/mL at 30 minutes and 35.3 ng/mL at 60 minutes. The patient was therefore diagnosed as CAH. Treatment was started with hydrocortisone and fludrocortisone and 1 g/day salt was added to the diet. A high level of aldosterone despite salt loss is not expected in CAH. We therefore performed renal ultrasonography to detect any renal anomaly that could cause a lack of response to aldosterone and found grade 2 hydronephrosis of the left kidney and bilateral grade 4-5 VUR on voiding cystogram. Amoxicillin prophylaxis was started. Genetic analysis revealed a heterozygous Q318X and homozygous IVS2 mutation of the 21-OH gene. Bilateral Teflon injection was performed for the VUR. The patient is currently 4 years old, is on hydrocortisone and fludrocortisone and is being followed-up without any problems.

**Patient 2**

A 35-day-old male baby born with a birth weight of 3500 g at term presented to the emergency service of our hospital with vomiting and failure to thrive. His weight was 3200 g, 

indicating a weight loss of 8% since birth. The patient's general condition was poor. Marked dehydration and mild scrotal hyperpigmentation were present. Laboratory results were as follows: Serum Na: 95 mEq/L (N: 135-143 mEq/L), K: 6.9 mEq/L (N: 3.5-5.5 mEq/L ), blood pH: 7.3, HCO3: 11.7 mmol/L, BUN: 26 mg/dL (0-10 mg/dL), creatinine: 0.47 mg/dL (0.3-1.2 mg/dL). Urinalysis was positive for nitrite and leukocytes and there was a growth of 100 000 colonies of Klebsiella on urine culture. Fludrocortisone and antibiotics were started together with intravenous fluid treatment. Renal ultrasonographic investigation for UTI showed prominent collective structures in the left kidney and grade 2 VUR on the voiding cystogram. Antibiotic prophylaxis was started. Hormonal evaluation revealed basal cortisol of 18 μg/dL, ACTH 89.5 pg/mL (N: 3-46 pg/mL), renin: 186 pg/mL (N: 2.4-37 pg/mL), and aldosterone level of 2 000 pg/mL (N:20-700 pg/mL). The preliminary diagnosis was PHA. The patient benefited from fludrocortisone treatment. Taking into account the high serum ACTH level, an ACTH stimulation test was performed to rule out CAH and the 17-OHP response was as follows: 27.6 ng/mL at 0 time, 44.1 ng/mL at 30 minutes and 41.2 ng/mL at 60 minutes. Hydrocortisone was added to the fludrocortisone and salt treatment. Genetic analysis revealed a large deletion of the 21-OH gene. The patient is currently 1 year old and is being followed-up without any problems on hydrocortisone, fludrocortisone and oral 1 g/day salt treatment. Age at presentation and laboratory findings of the patients are given in Table 1.

**Table 1:**
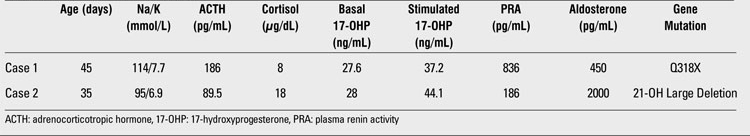
Laboratory findings of the patients

## DISCUSSION

Aldosterone is the primary mineralocorticoid hormone regulating serum electrolytes by enabling sodium absorption and potassium secretion at the distal tubule. Hyponatremia, hyperpotassemia, metabolic acidosis and hypovolemia develop due to renal salt loss in aldosterone deficiency. Isolated aldosterone deficiency is rare but can be seen in patients with the uncompensated salt-losing type of CAH and also in patients with autoimmune adrenalitis and congenital adrenal hypoplasia. PHA is a condition in which a high aldosterone level occurs together with signs and symptoms of aldosterone deficiency. PHA can be primary or secondary.  

Secondary PHA is limited to the kidneys and can develop due to drugs (cyclooxygenase inhibitors, angiotensinconverting enzyme inhibitors, potassium-sparing diuretics, trimethoprim, cyclosporine, etc.), congenital renal malformations, obstructive uropathy, VUR, UTI, tubulointerstitial nephritis, nephropathy in sickle cell anemia, renal amyloidosis, and  in multiple myeloma due to aldosterone insensitivity at the distal renal tubule (4).

Secondary pseudohypoaldosteronism has been reported in both sexes, but it can temporarily be seen due to congenital renal anomaly, obstructive uropathy, VUR and/or UTI in male infants under the age of 3 months, similar to our cases (2). A lack of response of the renal tubule to aldosterone is present in this condition. The mechanism of aldosterone resistance is not fully understood, but it becomes exaggerated in the presence of UTI (4).

A review of the 68 temporary PHA cases reported between 1983 and 2008 showed that 48 subjects had UTI together with obstructive uropathy, VUR or other urinary system abnormality, while 8 cases had obstructive uropathy or VUR without UTI. A UTI without obstruction was present only in 5 of the 68 patients. These findings indicate that PHA can develop in renal anomaly that does not cause obstruction, obstructive uropathy, VUR and/or UTI. Also PHA can develop even when there is only a UTI (5,6,7).

Melzi et al (8) found PHA in 17 of 50 infants (34%) aged 15 days to 15 months who had been  diagnosed as UTI. A urinary system anomaly was found in all 17 of these patients and all of these 17 patients had been diagnosed when they were younger than 3 months (8). The symptoms usually resolve by age 2 years with the completion of tubular maturation.  Secondary transient PHA resembles the SL-CAH clinically and biochemically and there are cases in the literature who have been initially diagnosed as CAH and started on glucocorticoid and mineralocorticoid treatment (4,9,10). The opposite situation applies to our cases and this has not been reported or noted until now. The secondary PHA in our cases was masking the CAH diagnosis. In the uncompensated salt-losing form of CAH, hyponatremia and hyperpotassemia occur due to aldosterone deficiency. Aldosterone is very high in the compensated salt-losing type and serum Na and K levels are usually normal as a result of no salt loss (3). The serum aldosterone levels were markedly high in both our patients, but the salt loss first pointed towards a diagnosis of PHA. However, the presence of VUR and UTI in the patients had led to a lack of aldosterone efficacy and masked the CAH diagnosis. Both our cases also had adequate basal cortisol levels. The CAH diagnosis was confirmed with the ACTH stimulation test and genetic analysis. 

In conclusion, the possibility of UTI and renal anomalies, which are seen commonly in infancy and cause a lack of aldosterone effect, should be kept in mind and the patients should be investigated accordingly. We suggest performing appropriate investigations and genetic studies for the differential diagnosis of CAH in the presence of renal problems or UTI in cases  presenting with hyponatremia, hyperpotassemia and high aldosterone levels. CAH should be ruled out in every case suspected of having PHA, especially in the presence of problems that may cause aldosterone resistance. 
